# Optimization of the microbiological quality control validation of corneal medium using a clinical *C. acnes* isolate

**DOI:** 10.1007/s10561-026-10211-9

**Published:** 2026-02-19

**Authors:** Philipp Maximilian Maurer, Gefion Franke, Johannes K. Knobloch

**Affiliations:** Institute for Medical Microbiology, Virology and Hygiene, Department Infection Prevention and Control, University Medical Center Hamburg-Eppendorf, Martinistr. 52, 20246 Hamburg, Germany

**Keywords:** Ph. Eur., Cornea, Sterility testing, Quality control testing, *C. acnes*

## Abstract

The microbiological quality of corneal tissue for transplantation must be monitored in a validated procedure complying with the European Pharmacopoeia (Ph. Eur.). An automated culture system is used to detect test strains in the presence of the medium. The Ph. Eur. mentioned *C. acnes* ATCC 11827 strain has allegedly been isolated from skin. This strain is known to display variable growth in routine matrix validation. As the Ph. Eur. also opens the use of clinical isolates, this study analyzed the use of clinical isolates for matrix validation.

We compared the time-to-detection (TTD) in absence and presence of cornea organ culture medium (cocm) of 24 *C. acnes* strains, preferring clinical isolates, in an automated culture system.

In the first step clinical strains (n=23) were examined, thus 4 slow-growing (TTD 305,1–336 h), 5 intermediate-fast-growing (TTD 250,1-305 h), 5 fast-growing strains (TTD 164,3–250 h) and nine non-blood-culture-detectable strains (TTD>336 h) were identified. Seven strains showed a reproducible growth signal in cocm-absence. In presence of cocm, three strains (CA02, CA10, CA22) showed a reproducible growth signal, while the reference strain was not blood-culture-detectable (bcd) in four of five experiments. The strain with the preferred growth characteristics (DSM 117854) was tested according to the Ph. Eur. conditions and showed bcd growth in all measurements (n=5) in presence of medium, in contrast to the reference strain.

The use of a clinical isolate of *C. acnes* could improve matrix validation of cocm quality control testing and thus the care of cornea-transplant-patients. *C. acnes* DSM 117854 provides an isolate that promises more reliable results in matrix validation.

**Supplementary Information:**

The online version contains supplementary material available at 10.1007/s10561-026-10211-9.

## Introduction

The European Pharmacopoeia (Ph. Eur.) (European Pharmacopoeia, 10th edition 2019, English 2019) is the primary source of official quality control standards of pharmaceutical products in Europe. According to the Ph. Eur., the microbiological quality of corneal tissue for transplantation must be monitored in a validated procedure. Concerning the safety of tissue preparations, the monograph 2.6.27, Microbiological examination of cell-based preparations, specifically demands microbiological sterility testing to prove the absence of potential harmful contaminants. In the case of tissue preparations from which a representative sample cannot be taken without loss of function, an indirect sample, for example the organ culture medium can also be analyzed. Cornea organ culture medium (cocm) containing antibiotics is the storage medium of choice in most European cornea banks (Armitage and Easty 1997) and is used for sterility testing as an indirect sample. Automated methods are listed first by the Ph. Eur. for these mandatory sterility tests. As a further growth based method, classic growth culture testing is possible (European Pharmacopoeia, 10th edition 2019, English 2019; Paul-Ehrlich-Institut 2022). However, this approach requires a considerably higher, often disproportionate amount of internal laboratory quality control. In addition, the method is more prone to errors when using cloudy or colored solutions, which makes it difficult to assess whether growth is present. Therefore, the use of classical cultural procedures is uncommon. Additionally, the Ph. Eur. is also open to the use of direct detection methods for microorganisms, such as molecular detection using polymerase chain reaction. (PCR). However, the Ph. Eur clearly states the limitations of using these methods: a positive PCR signal may be obtained during testing, but the organisms may not be viable, and PCR may be less sensitive than culture. For the use of these methods, the Ph. Eur. specifically mentions sterility testing of products with a short shelf life (European Pharmacopoeia, 10th edition 2019, English 2019). In Europe, the use of blood cultures as an automated detection method is widely used as a rapid and sensitive method, for small amounts of microbial contamination in cocm, a product with a shelf life of approximately 4 weeks (Thuret et al. 2002; Mistò et al. 2020). However, even with highly sensitive methods some difficult to cultivate organisms might be missed and all methods for detecting potential contaminants must always strike a balance between sensitivity and speed in order to achieve a standardized result.

In automated procedures, the medium validation is done by the manufacturers, so that the testing laboratory only has to verify the inhibition of the antibiotics contained in the cocm as matrix validation (Paul-Ehrlich-Institut 2022). In this matrix validation of the microbiological detection method according to Ph. Eur., the detection of a defined inoculum (maximum 100 colony-forming units) of certain fungi, aerobic and anaerobic bacteria must be demonstrated in the presence of the organ culture medium to be tested. *Cutibacterium acnes* represents a microaerophilic microorganism that must be part of the matrix validation according to monograph 2.6.27, specifically named *C. acnes* ATCC 11827 or a clinical isolate.

*C. acnes* is known to be a difficult to cultivate species, for which not all strains can be grown even in media without inhibitory additives (Jeverica et al. 2020). It has also been shown that human-derived cells and matrix proteins can inhibit their growth (Arlt et al. 2017). Since many bacteria can achieve a viable but non-culturable status (VBNC) and cannot be detected immediately using cultural methods (Pazos-Rojas et al. 2023), reliably culturable strains should be used for quality control to enable standardised quality control. In our routine matrix validation of the cocm sterility test, a semiautomated blood culture bottle system, approved by the German authorities, was used. *C. acnes* ATCC 11827 was repeatedly not blood-culture-detectable (bcd) during the preset time period of 14 days (336 h). Besides the mentioned *C. acnes* ATCC strain, the Ph. Eur. allows the use of other strains for validation. To improve the matrix validation of our sterility testing, we compared the time to detection (TTD) in blood culture bottles of 23 *C. acnes* strains of different origin, preferring clinical isolates.

## Material and Methods

### Strains

Nineteen of the strains used originate from tissue obtained intraoperatively of infectious sites as prosthetic joint infections, osteomyelitis or endocarditis. Four of the strains were derived from human skin and were selected as controls. The reference strain used was ATCC 11827, which is supposed to originate from the human skin (Miquel et al. 2022). All strains were stored using lyophilized glass beads (CRYOBANK, Mast Group Ltd., Bootle Mersey-side, UK). Prior the experiments, the strains were recultivated on Schaedler Agar 5% sheep blood plus (bioMérieux, Marcy l’Etoile, France) for 72 h under anaerobic growth conditions at 37 °C.

**Table 1:** Origin of the strains used

Strain	Isolate origin	MIC_penicillin_ (µg/ml)	Strain	Isolate origin	MIC_penicillin_ (µg/ml)	Strain	Isolate origin	MIC_penicillin_ (µg/ml)	Strain	Isolate origin	MIC_penicillin_ (µg/ml)
CA01	Clinical		CA07	Clinical		CA13	Clinical		CA19	Clinical	
CA02^a^	Clinical	0,032	CA08	Clinical		CA14	Clinical	0,016	CA20	Skin	
CA03	Clinical	0,064	CA09	Clinical		CA15	Clinical		CA21	Skin	
CA04	Clinical	0,032	CA10	Clinical	0,016	CA16	Clinical		CA22	Skin	0,032
CA05	Clinical		CA11	Clinical		CA17	Clinical		CA23	Skin	
CA06	Clinical		CA12	Clinical	0,032	CA18	Clinical		ATCC 11827	Skin	0,016

MIC, Minimum inhibitory concentration. ^**a**^This isolate was deposited as C. acnes DSM 117854 at DSMZ.

### Growth characteristics of *C. acnes* in blood culture bottles

Fresh cultivated colonies were resuspended in isotonic saline solution and the concentration was adjusted to a turbidity of 0,5 McFarland, using the bioMérieux DensiCHEK Plus densitometer (bioMérieux, Marcy l’Etoile, France). Subsequently, dilutions were prepared with saline solution to approximately 1000 colony forming units (CFU) / mL. To verify the actual inoculum used, 100 µL of each stock solution were spread in duplicate on Schaedler Agar 5% sheep blood plus (bioMérieux, Marcy l’Etoile, France). After an anaerobic incubation for at least 48 h, the CFU were counted and the arithmetic mean of both measurements was calculated.

To evaluate the efficacy of cultivating *C. acnes* in the presence of cornea organ culture medium (cocm) TISSUE-C (AL.CHI.MI.A., Ponte San Nicolò, Italy), the semiautomated blood culture system BD BACTEC FX and BD BACTEC Plus Aerobic/F and Anaerobic/F blood culture bottles containing antibiotic-inactivating resins (Becton Dickinson, Heidelberg, Germany) were used. To prepare blood culture bottles without cocm, for each experimental run 100 µL stock solution of the respective *C. acnes* strain to be tested plus 10 mL isotonic saline solution were injected, followed by incubation in the blood culture system. To prepare blood culture bottles with cocm, for each experimental run 9 mL cocm plus 1 mL Penicillinase (Penicillinase Concentrate, 10 Million Units per mL, Becton Dickinson) were injected into an anaerobic blood culture bottle, followed by a 1 h incubation at room temperature. Afterwards 100 µL of the respective *C. acnes* stock, was added, followed by incubation in the blood culture system.

The incubation temperature of the system was 36–38 °C. The system uses a fluorescin indicator system reacting with microbial metabolites for continuous detection of microbiological growth. A growth signal was recorded automatically or the incubation lasted a maximum of 336 hours. After a growth signal detection, 100 µL of the bottle content was subcultured on the appropriate agar plates. The negative controls and the bottles without blood-culture-detectable growth spiked with cocm and *C. acnes* were subcultured after the set maximal incubation time. Schaedler agar 5% sheep blood plus (bioMérieux) was used for the subcultivation of the anaerobic bottles by incubation under anaerobic growth conditions at 37 °C for 72 h. Columbia Sheep blood Agar plus (bioMérieux) was used for the subcultivation of the aerobic bottles by incubation under aerobic growth conditions at 37 °C for 48 h, respectively. Species identification was confirmed by matrix-assisted laser desorption ionization time-of-flight (MALDI-TOF) mass spectrometry (MALDI Biotyper MBT SMART IVD, Bruker, Bremen, Germany). Sterility testing in aerobic and anaerobic blood culture bottles of isotonic saline solution (0.9% NaCl; B. Braun Melsungen AG, Germany) and cocm was carried out in parallel for each run of the experiment. Five repetitions of each experiment were performed to verify the reproducibility of the procedure and allow potential random fluctuations to be identified.

### Antimicrobial susceptibility testing

The minimum inhibitory concentration (MIC) of penicillin for eight bcd strains (ATCC 11827, CA02, CA03, CA04, CA10, CA12, CA14. CA22) used for experiments in presence of cocm was determined, using ETEST (bioMérieux) on brucella agar (Becton Dickinson) with an inoculum of McFarland 1 and an anaerobic incubation time of 18 h.

### Deposition of the *C. acnes* DSM 117854

The strain CA02 with the desired growth characteristics was sent to deposit in the open collection of the Leibniz Institute DSMZ - German Collection of Microorganisms and Cell Cultures GmbH as *C. acnes* DSM 117854. The initial sent strain (CA02) as well as the retransmitted strain by DSMZ (DSM 117854) were analyzed using next-generation sequencing as recently described (Carlsen et al. 2022): DNA was extracted from a pure culture using the MagNA Pure 96 system (Roche Diagnostics, Rotkreutz, Switzerland), according to the manufacturers’ guidelines. DNA-quantity was measured via fluorescent dye photospectrometry using the Qubit 3.0 device (ThermoFisher Scientific, Waltham, USA). An external provider carried out sequencing. The gained raw-reads of both strains were published in the National Center for Biotechnology Information (NCBI) GenBank under Biosample accession number SAMN46824307 (CA02) and SAMN46824308 (DSM 117854).

To compare both strains, *ad hoc* core genome Multi Locus Sequence Typing (cgMLST) scheme was calculated in the Ridom SeqSphere+ (version 10.0.4) (Ridom, Münster, Germany) using the seed genome of *C. acnes* KPA171202 (Accession nr.: NC_006085.1; core genome: 2003 alleles).

## Results

For a preliminary investigation of the general detection rate in blood culture bottles and for identifying fast growing isolates a single experiment to determine the time to detection (TTD) of 23 non-reference *C. acnes* strains was conducted. The TTD showed a spectrum from 164,3 h to no blood-culture-detectable (bcd) growth within the set time period of 336 h [Supp. Fig. 1]. For all experiments described negative controls remained without bcd growth in the preset time period of 336 h.

Nine of the 23 strains examined, did not show a growth signal during the preset 336 h (CA01, CA05, CA07, CA08, CA09, CA13, CA17, CA18, CA19).

The five strains with the shortest TTD (CA22: TTD= 164,3 h, CA03: TTD= 193,5 h, CA02: TTD= 212,2 h, CA14: TTD= 241,9 h, CA04: TTD= 249,5 h,) and the four bcd strains with the highest TTD (CA23: TTD= 306,1 h, CA10: TTD= 311,3 h, CA20: TTD= 321,1 h, CA12: TTD= 333 h,) were tested further for reproducibility by repeating the measurement. Seven *C. acnes* strains showed a reproducible TTD in presence of isotonic saline solution (CA02, CA03, CA04, CA10, CA12, CA14, CA22) in a total of 5 experiments (n=5) [Supp. Fig. 2]. The fast growing strains CA02 (TTD_mean_=212,72 h; SD=9,54; detection rate=100%), CA03 (TTD_mean_=198,52 h; SD=32,25; detection rate=100%), CA04 (TTD_mean_=242,48; SD=10,94; detection rate=100%), CA14 (TTD_mean_=240,84 h; SD=35,39; detection rate=100%) and CA22 (TTD_mean_=190,56 h; SD=16,86; detection rate=100%) showed a shorter TTD_mean_ than the slow growing strains CA10 (TTD_mean_=308 h; SD=23,87; detection rate=100%) and CA12 (TTD_mean_=316,05 h; SD=21,48; detection rate=80%). The two other strains had a standard deviation of more than 40 (CA23; TTD_mean_=279,48 h; SD=46,17; detection rate=100%), or were not bcd in four of five measurements (CA20; TTD_1_= 321,1 h; detection rate=20%) respectively. The latter two were excluded from further examination. Content of each bottle showing bcd growth was subcultured on Schaedler agar under anaerobic conditions and growth of *C. acnes* was confirmed by identification using MALDI-ToF. There were no experiments with confirmed contamination by detection of bacterial species other than *C. acnes*.

**Fig. 1:**
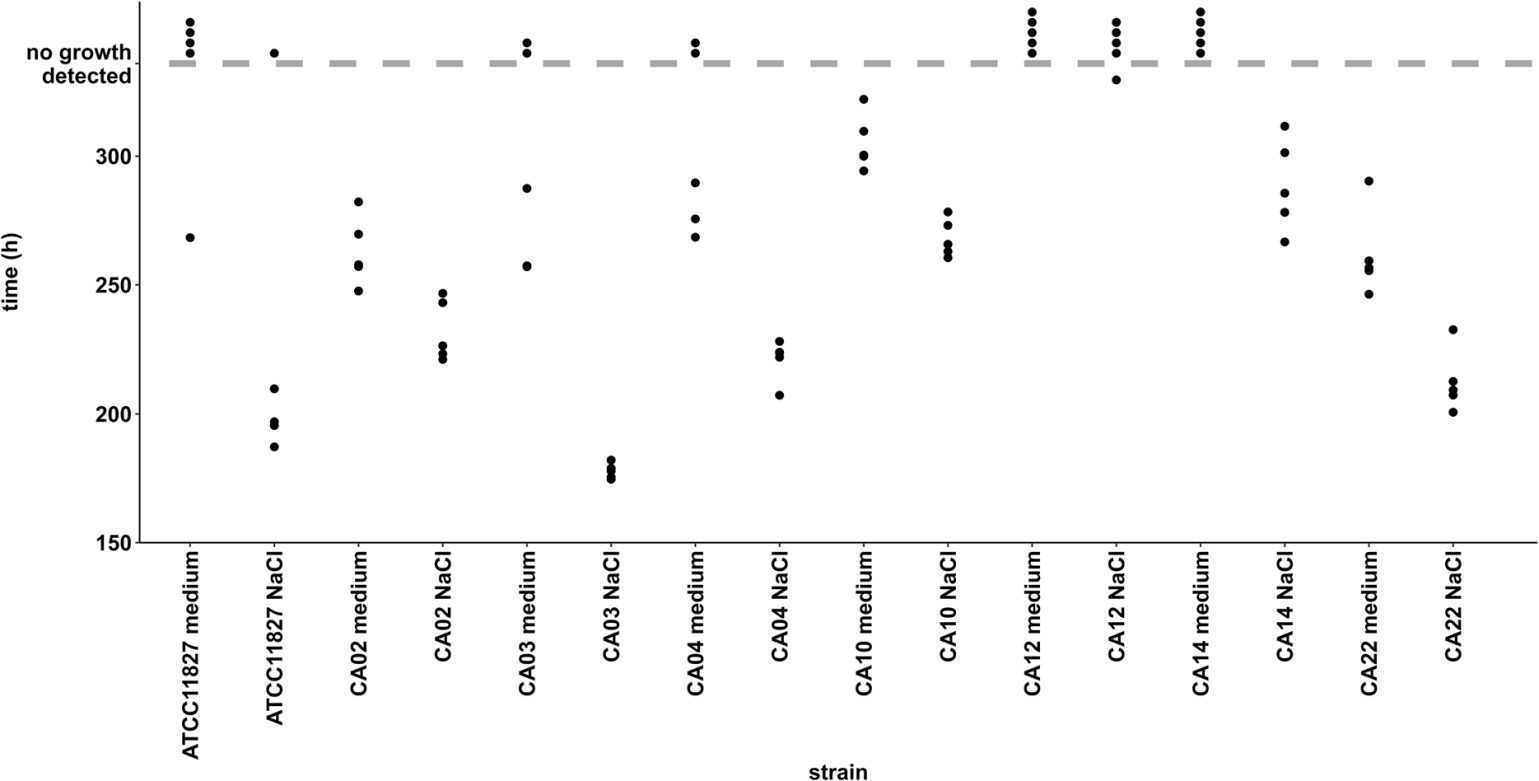
Dots representing the time to detection (TTD) of seven clinical strains and the ATCC 11827 *C.* *acnes* strains in anaerobic blood culture bottles with and without addition of medium. The dashed line represents the preset incubation time period of 336 hours. Dots above the dashed line represent experiments with non-bcd results

In order to compare the TTD in presence and absence of cornea organ culture medium (cocm), non-reference strains CA02, CA03, CA04, CA10, CA12, CA14, CA22, as well as reference strain ATCC 11827 were inoculated in anaerobic blood culture bottles with and without the addition of cocm plus penicillinase and incubated, for a total of 5 experiments. In addition, the susceptibility of these strains to penicillin was measured, and a minimum inhibitory concentration (MIC) range of 0.016 µg/mL to 0.064 µg/mL was determined (Table 1). All tested *C. acnes* strains showed a longer TTD_mean_ with a higher SD in presence of medium [Fig. 1]. CA02 and CA22 showed the shortest TTD_mean_ in presence of cocm (CA02_medium_: TTD_mean_=262,89 h; SD=13,35; detection rate=100%; CA22_medium_: TTD_mean_=261,63 h; SD=16,77; detection rate=100%) and were bcd in all measurements. CA10 showed a longer TTD_mean_ in presence of cocm (CA10_medium_: TTD_mean_=305,28 h; SD=10,89; detection rate=100%) and was bcd in all measurements. CA03 and CA04 were not bcd in presence of cocm in two out of five measurements (CA03_medium_: TTD_mean_=267,3 h; detection rate=60%; CA04_medium_: TTD_mean_=277,92 h; detection rate=60%), while both strains were bcd in all parallel measurements in absence of cocm. CA12 and CA14 were not bcd in all five measurements in presence of cocm (detection rate=0%). In the parallel approach in absence of cocm, a growth signal was bcd in all five (CA14_NaCl_: detection rate=100%), or in one out of five measurements (CA12_NaCl_: detection rate=20%). The Ph. Eur. suggested *C. acnes* ATCC 11827 was bcd in presence of cocm in one out of five measurements (TTD_1_=268,35 h; detection rate=20%), while it was bcd in four of five measurements in absence of cocm (ATCC 11827_NaCl_: TTD_mean_= 197,33 h; SD_mean_= 9,25; detection rate=80%). The contents of each bottle that showed bcd growth or had reached the end of the incubation period were subcultured. The growth of *C. acnes* in the subculture was confirmed by MALDI-ToF, with the exception of CA03 and CA04 in a single experimental run. After the incubation in blood culture bottles containing cocm, both strains showed no growth of *C. acnes* or any other species in the subculture.

Regarding the count of colony-forming units (CFU), which was used for the repeated measurements of TTD in presence (n=5) and absence (n=10) of culture medium, the mean concentration used was 107,03 CFU/bottle and SD=45,42 [Supp. Fig. 3].

**Fig. 2:**
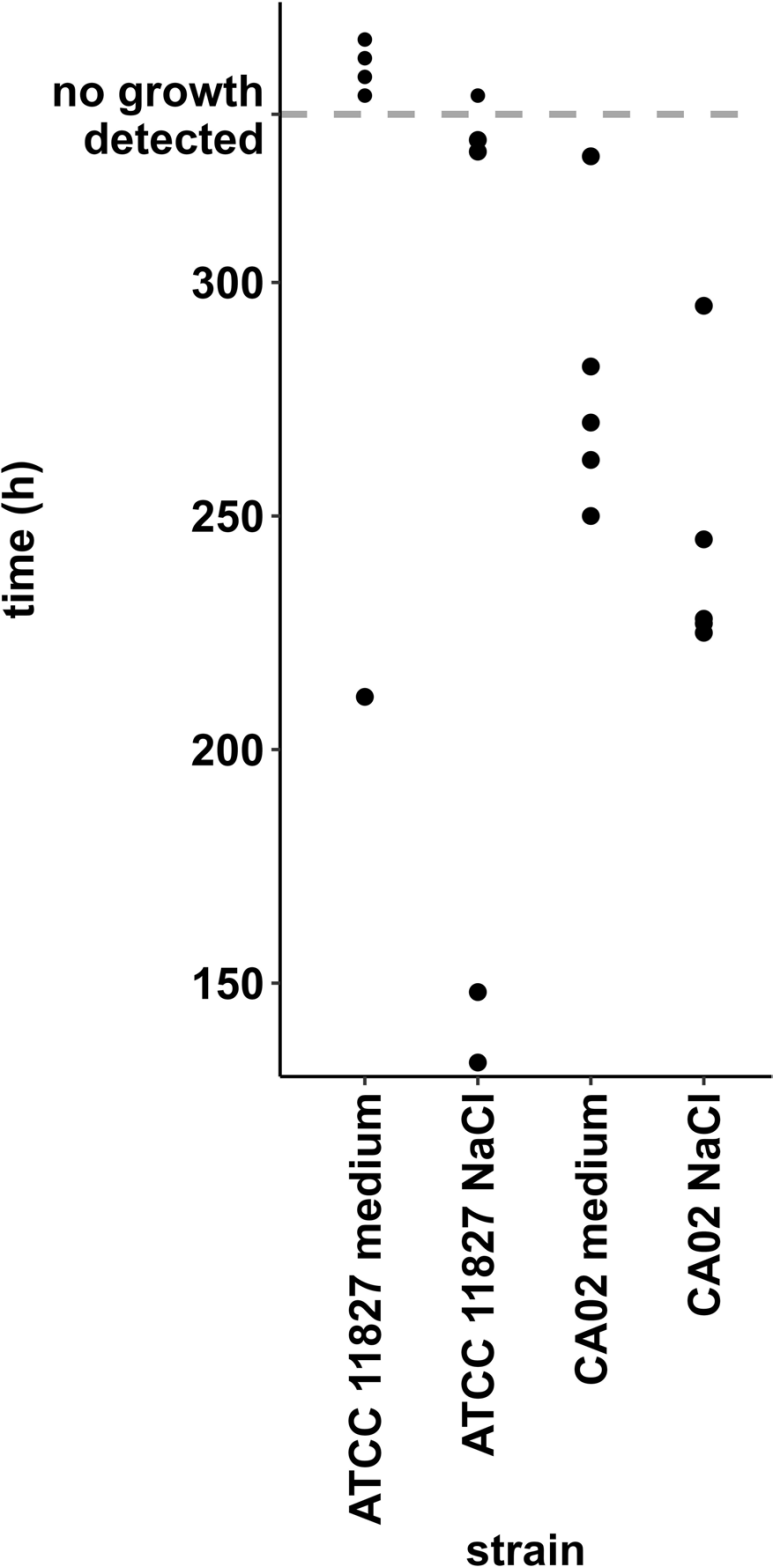
Dots representing the time to detection (TTD) of a maximum of 100 colony-forming units (CFU) of *C. acnes* CA02, the strain with the favorable growth characteristics, in comparison to the ATCC 11827 strain (spreading width = 105–625 CFU), in anaerobic blood culture bottles with and without addition of cornea organ culture medium. There were a total of five independent measurements per strain. The dashed line represents the preset incubation time period of 336 hours

CA02 was chosen as the strain with the favorable growth characteristics for further examination, due to the lower SD and repeatable growth detection signal in all five measurements in presence of cocm. To compare the TTD of CA02 under the conditions required by Ph. Eur., the used CFU-count of the stock solution was precisely adjusted to 10-100 CFU/100µL, blood culture bottles with or without the addition of cocm were prepared and incubated. In a parallel approach, ATCC 11827 was tested. The CFU-count of CA02 (mean=62,2 CFU/ bottle, spreading width= 43 CFU/ bottle - 82 CFU/ bottle) was lower than of the ATCC-strain (mean=363,3 CFU/bottle, spreading width = 105–625 CFU/bottle) [Supp. Fig. 4]. Regardless of the additives, CA02 was bcd in the five experimental runs [Fig. 2]. The TTD was repeatedly longer in the presence of cocm (CA02_medium_: TTD_mean_=278,2 h, SD=29,67; detection rate=100%) than in its absence (CA02_NaCl_: TTD_mean_=244 h, SD=29,61; detection rate=100%). In comparison, ATCC 11827 was bcd in presence of cocm in one out of five (ATCC 11827_medium_: TTD_1_=211,28 h; detection rate=20%) and in absence of cocm in four out of five measurements (ATCC 11827_NaCl_: TTD_mean_=230,5 h, detection rate=80%). Terminal subculture under anaerobic conditions of *C. acnes* inoculated bottles without bcd growth resulted in growth of *C. acnes*.

CA02 was transferred to the DSMZ strain collection and deposited as DSM 117854. Core genome Multi Locus Sequence Typing (cgMLST) using an *ad hoc* cgMLST scheme was performed and showed no difference between the alleles of CA02 and DSM 117854.

## Discussion

Microbial contamination of cornea grafts is a rare but potentially graft-threatening event (Chen et al. 2015). Microbial testing of cornea grafts must be a validated procedure according to the guidelines of the European Pharmacopoeia (Ph. Eur.). Since the culture medium validation is carried out by the culture medium manufacturer, the Ph. Eur. requires proof of the cultivability of individual defined reference species in the presence of the cornea organ culture medium (cocm) as part of matrix validation in the laboratory, which performs sterility testing (European Pharmacopoeia, 10th edition 2019, English 2019; Paul-Ehrlich-Institut 2022). The use of blood culture bottle enrichment as automated cultivation method for sterility testing is common (Thuret et al. 2002; Thomasen et al. 2015; European Pharmacopoeia, 10th edition 2019, English 2019). Most of the genera, which need to be tested, show a growth signal during the first 72h in the blood culture enrichment method depending on the matrix, incubation temperature and test strain used (Thomasen et al. 2015). In contrast *C. acnes* requires a longer cultivation time until a growth signal is detected (Lotfi et al. 2020; Herrlinger et al. 2021). One of the reasons described for the late blood-culture-detectable (bcd) growth, is the overall low metabolic activity of slow-growing *C. acnes.*

The observed non-reliability of Ph. Eur. suggested reference strain *C.* *acnes* ATCC 11827 is consistent with the result of prior matrix validation procedures in our laboratory, showing no bcd growth in presence of cocm in several independent experimental series. The general difficulties to cultivate *C. acnes* was confirmed in this study, as several isolates tested in this study displayed no detectable growth, even in absence of cocm. Since in general not all viable microorganisms can be detected using cultural methods under laboratory conditions, strains that can be reliably cultivated should be used for quality control and matrix validation.

To identify such a strain, the time to detection (TTD) in anaerobic blood culture bottles of different *C. acnes* strains of clinical sample origin was measured and showed a broad variability even without further, potentially inhibiting factors [Supp. Fig. 1]. This variability was reproducible for the strains examined further in absence of cocm, which suggests that *C. acnes* strains show repetitively strain specific growth rates [Supp. Fig. 2]. This result is consistent with the literature, where studies comparing the TTD of different *C. acnes* strains in absence of potentially inhibiting factors in blood culture bottles showed varying results. By comparing the TTD of *C. acnes* strains, some of which are associated with orthopedic-related infections and originate from different clonal complexes, a study showed that isolates are bcd after a clonal-complex (CC)-unique time period (n = 72, growth rate 97,2%). The measured TTD_median_ varied from approx. 6 days (CC18) to approx. 3 days (CC53) (El Sayed et al. 2021). In a second study using the same bottles as in our study (BD Bactec Plus Anaerobic/F bottles) and an inoculum according to Ph.Eur. (CFU-count=15 CFU/bottle), five strains of the examined 26 infection-site-origin *C. acnes* isolates, showed a growth signal during 14 days. The TTD_mean_ of those five strains was approx. 11 days (detection rate=19%) (Rentenaar et al. 2018). Interestingly, the TTD_mean_ of the 26 clinical *C. acnes* strains was shorter (TTD_mean_= approx. 5 days, detection rate=96%, CFU-count=15 CFU/bottle) in a different type of blood culture bottle (BD Bactec Lytic/10 Anaerobic/F) (Rentenaar et al. 2018). These results are consistent with another study, which measured the TTD of 99 clinical *C. acnes* strains associated with orthopedic related infections in two different semiautomated systems with the according bottles (CFU-count= approx. 100 CFU/bottle). A shorter TTD_mean_ and a higher detection rate of *C. acnes* were observed when using the BD Bactec Lytic/10 Anaerobic/F bottles (TTD_mean_= approx. 5 days, detection rate 92%) instead of the BD Bactec Plus Anaerobic/F bottles (TTD_mean_= approx. 10 days, detection rate 42%) (Jeverica et al. 2020). In conclusion, different strains of clinical origin exhibit different TTD in blood cultures, even if no other growth-inhibiting additives are present, depending on the used blood culture bottles, the semiautomated system and the temperature.

With regard to the TTD measurements in the presence of cocm, the antibiotic additives streptomycin and penicillin are a possible interference factor for the detection of bacteria in sterility testing. For this reason, penicillinase is added into the blood culture bottles as part of sterility testing, as described previous (Thomasen et al. 2015). In order to exclude differences in their phenotypic antibiotic susceptibility as an explanation for the late or absent growth in the presence of cocm, the minimum inhibitory concentration (MIC) for penicillin was measured. The MIC_penicillin_-range (0,016 μg/mL - 0,064 μg/mL, Tab. 1) was within the sensitivity range (Susceptible ≤ 0,06g/mL) according to the European Committee on Antimicrobial Susceptibility Testing (EUCAST) (European Committee on Antimicrobial Susceptibility Testing 2025). Consequently, a correlation between an increased MIC_penicillin_ in combination with an incomplete inactivation of the antibiotic additives as the cause of the difference in blood culture detection is unlikely.

With regard to other cell preparations, a study focusing matrix-validation showed the matrix- and blood-culture-bottle-depended growth of *C. acnes*. In the presence of hematopoietic stem cells (HSC), C. acnes could only be detected in one of the three blood culture bottles tested. The authors attribute this result to inactivation of *C. acnes* by HSC (Arlt et al. 2017). The German authorities expressly refer to the possible bacterial inactivation by the product. They therefore recommend the use of bacterial strains for HSC sterility testing matrix validation purpose that have been previously identified by routine sterility tests (Paul-Ehrlich-Institut 2022). The underlying reason for this recommendation might be different bacterial robustness to environmental influences, such as growth inhibiting factors in the matrix.

For many bacteria the viable but non-culturable status (VBNC) is described as a mechanism that increases the bacterial robustness for survival in a hostile environment. This status makes bacterial detection difficult using cultural methods (Pazos-Rojas et al. 2023). There are no reports in the literature that *C. acnes* transitions to this status. Therefore, further research is needed to determine whether *C. acnes* can transition and which other mechanisms meditate *C. acnes* bacterial robustness. Interestingly, VBNC status as a difficulty in microbiological quality control is already in focus of certain studies. However, there are no studies focusing pharmaceutical sterility testing. The studies primarily address water microbiological quality control and the effects, that promote bacteria to transition to the VBNC-status (Lin et al. 2017; Ng et al. 2021). In summary, a single reason for the different TTD_NaCl_ and TTD_medium_ between the examined strains was not identified, a difference of bacterial robustness could be the explanation.

CA02 was identified as a strain with constant growth detection needed for reliable quality control, showing a TTD_mean_ in presence of cocm of approx. 11 days and a TTD_mean_ in cocm presence and Ph. Eur. according CFU-count (10-100 CFU/bottle) of approx. 12 days (detection rate 100% in both experimental runs). This result is consistent with one of the two studies focusing on TTD of *C. acnes* in presence of cocm, in which a TTD_mean_ of approx. 9 days in presence of the cocm used was measured (detection rate: 100%, n=4, temperature: 32 °C, BacT/Alert test system with the according bottles) for *C. acnes* ATCC 6919 (Thomasen et al. 2015). Regarding the other study focusing TTD-measurements in cocm-presence, *C. acnes* strain ATCC 6919 was detected in 10/12 measurements (cumulative detection rate approx. 83,3%), depending on the medium used (one femoral head medium, two different cocm) and on the incubation temperature (room temperature, 30 °C, 35 °C). The shortest TTD_mean_ was measured at 35 °C in femoral head medium (Herrlinger et al. 2021). It seems possible that the optimum temperature for growth of the skin commensal *C. acnes* ATCC 6919 is not at 37 °C as in our study, but at 30 °C or at 35 °C. In many laboratories, the same blood culture system is used for sterility testing and patient diagnostics at the same time, the temperature cannot be adjusted for validation. The Ph. Eur. is open for several different temperature settings, 35–37°C for anaerobic incubation of blood culture bottles is common (European Pharmacopoeia, 10th edition 2019, English 2019), matching the temperature of 36 °C +/− 1 °C used for patient-centered blood culture diagnostics (Expertengremium Mikrobiologisch-Infektiologische Qualitätsstandards 2007a, 2007b). Moreover, a *C. acnes* strain, which grows at 37 °C appears to be more of a human pathogen. The effect of temperature was not part of our study nor were the differences between different organ culture media. With regard to the both studies focusing on TTD of *C. acnes* in presence of cocm, a comparison of the results is only possible with limitations due to the use of different blood culture bottles, semi-automated blood culture systems, the media used, *C. acnes* strains and the incubation temperature. ATCC 6919 originates from the skin (Thomasen et al. 2015; Herrlinger et al. 2021), so its use as indicator organism representing strains of ocular infections to improve the validation of cocm sterility testing is therefore questionable.

Due to the late or absent detection of ATCC 11827, the incubation period for blood cultures for cocm sterility testing had already been extended to 14 days in our laboratory. The maximum storage interval of cocm is limited to four weeks (Mistò et al. 2020), meaning a further extension of the incubation period would shorten the time period for possible reimplantation. Based on our results, using strain CA02 for matrix validation will result in reliable quality control without further extension of the incubation period and reimplantation of the graft remains possible within the current time frame. The identified strain CA02 was submitted to the DSMZ under the designation DSM 117854. Given the possibility of using other *C. acnes* strains for quality control in microbial testing, we propose the use of this well-characterized strain. Nevertheless, terminal subcultivation seems to be inevitable in the sterility testing of cocm to exclude bacterial contamination, due to the successful cultivation of *C. acnes* from initially *C. acnes-*spiked bottles containing cocm, which did not show bcd growth. Further research is needed in pharmaceutical sterility testing, focusing on the detection of potentially harmful bacteria that are difficult to culture due to their slow growth or transition to VBNC status.

## Supplementary Information

Below is the link to the electronic supplementary material.

Supplementary file1 (PDF 158 KB)

Supplementary file2 (PDF 161 KB)

Supplementary file3 (PDF 207 KB)

Supplementary file4 (PDF 137 KB)

Supplementary file5 (JPG 43 KB)

## Data Availability

Sequence data that support the findings of this study have been deposited in the National Center for Biotechnology Information (NCBI) GenBank with the Biosample accession number SAMN46824307 (CA02) and SAMN46824308 (DSM 117854)
