# Supplementary material for: Optimization of the microbiological quality control validation of corneal medium using a clinical C. acnes isolate
Source: Cell Tissue Bank. 2026 Feb 19;27(1):12. doi: 10.1007/s10561-026-10211-9 (PMC12920408; doi:10.1007/s10561-026-10211-9)
Supplement: Supplementary file 1 — Supplementary file1 (PDF 158 KB) [file 10561_2026_10211_MOESM1_ESM.docx]

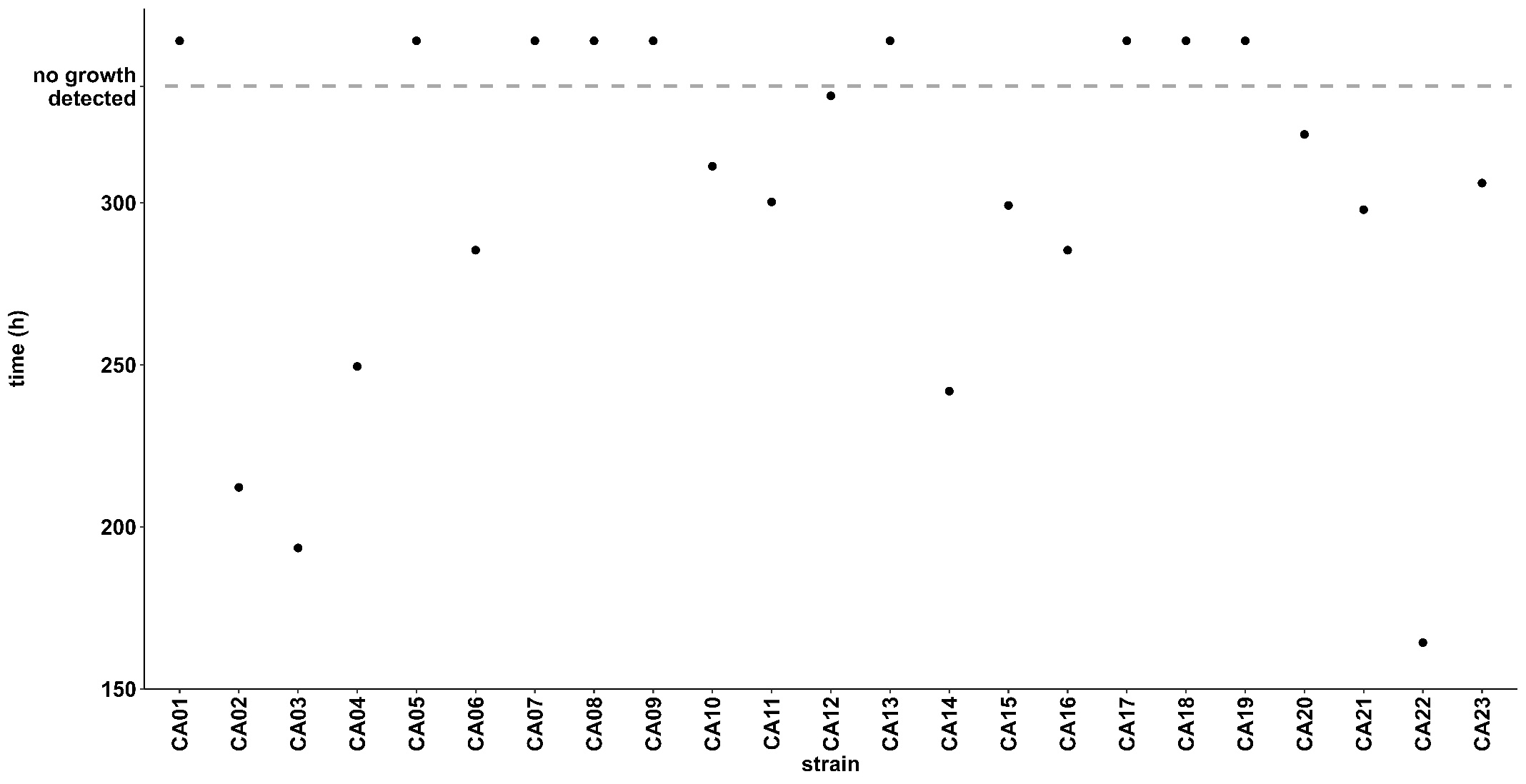


**Supp. Fig. 1** Comparison of the time to detection (TTD) in a semiautomated blood culture system of 23 *C. acnes* strains in anaerobic blood culture bottles, without addition of cornea organ culture medium. A black spot represents the time to detection of each strain. The dashed line represents the pre-set incubation time period of 336 hours.
