# Supplementary material for: Optimization of the microbiological quality control validation of corneal medium using a clinical C. acnes isolate
Source: Cell Tissue Bank. 2026 Feb 19;27(1):12. doi: 10.1007/s10561-026-10211-9 (PMC12920408; doi:10.1007/s10561-026-10211-9)
Supplement: Supplementary file 2 — Supplementary file2 (PDF 161 KB) [file 10561_2026_10211_MOESM2_ESM.docx]

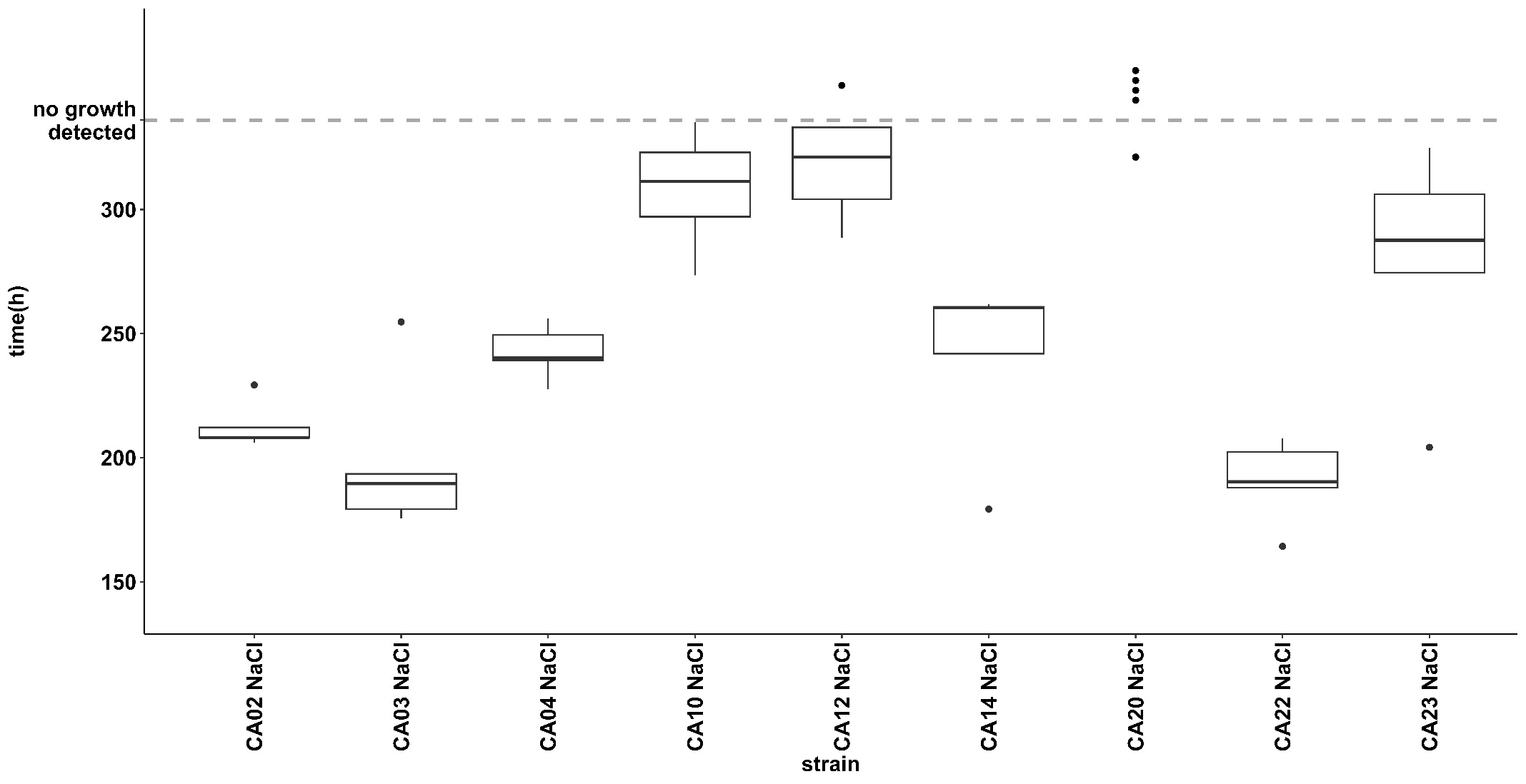


**Supp. Fig. 2** Box plots showing the time to detection of nine *C. acnes* strains in anaerobic blood culture bottles with the addition of isotonic saline solution (NaCl). A total of five independent measurements were performed per strain. The dashed line represents the pre-set incubation time period of 336 hours.
