# Supplementary material for: Optimization of the microbiological quality control validation of corneal medium using a clinical C. acnes isolate
Source: Cell Tissue Bank. 2026 Feb 19;27(1):12. doi: 10.1007/s10561-026-10211-9 (PMC12920408; doi:10.1007/s10561-026-10211-9)
Supplement: Supplementary file 3 — Supplementary file3 (PDF 207 KB) [file 10561_2026_10211_MOESM3_ESM.docx]

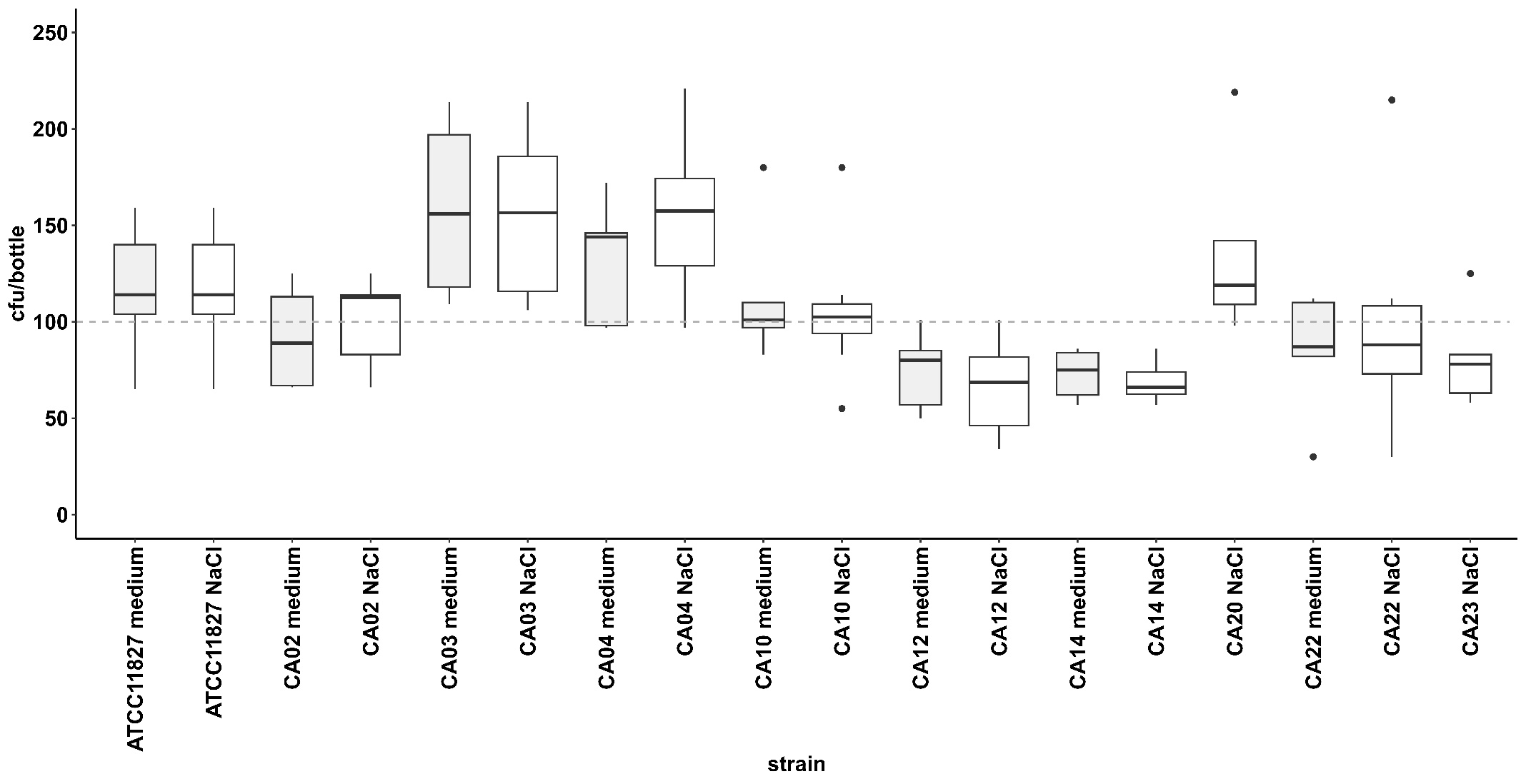


**Supp. Fig. 3** Box plots showing the distribution of colony-forming units (CFU) used to compare measurements with cornea organ culture medium and those with NaCl for nine clinical strains and the ATCC 11827 strain. The wide box plots represent n=10 and the narrow ones n=5 measurements, respectively. The dashed line represents the maximum of 100 CFU/bottle, required by the Ph. Eur..
