# Supplementary material for: Optimization of the microbiological quality control validation of corneal medium using a clinical C. acnes isolate
Source: Cell Tissue Bank. 2026 Feb 19;27(1):12. doi: 10.1007/s10561-026-10211-9 (PMC12920408; doi:10.1007/s10561-026-10211-9)
Supplement: Supplementary file 4 — Supplementary file4 (PDF 137 KB) [file 10561_2026_10211_MOESM4_ESM.docx]

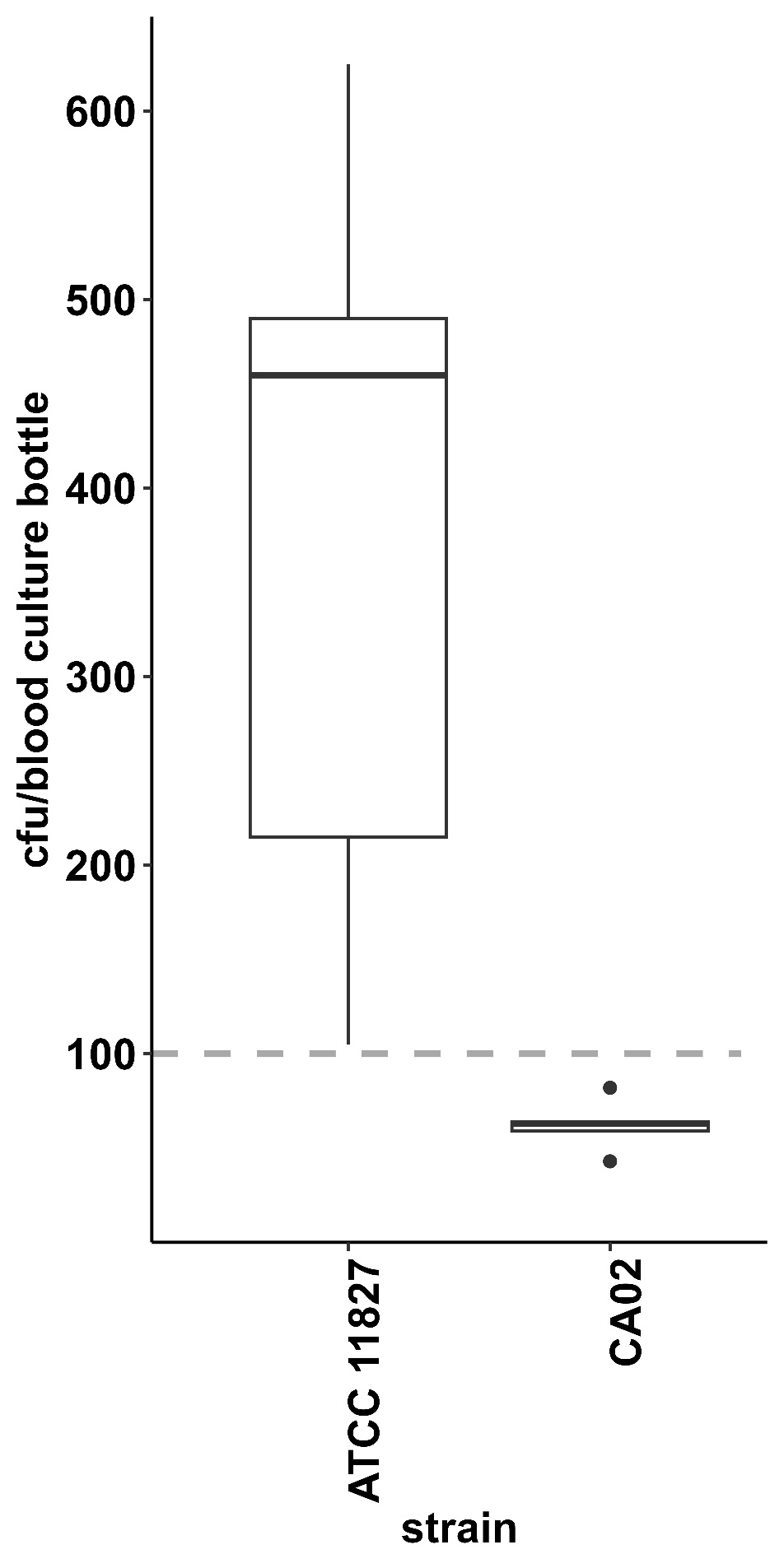


**Supp. Fig. 4** Box plots showing the distribution of colony-forming units (CFU) used for the TTD-comparison of CA02 and the ATCC 11827 strain in five independent experimental runs under Ph. Eur. conditions for CA02. The dashed line represents the maximum of 100 CFU/bottle per measurement, required by the Ph. Eur. .
