# Supplementary material for: Optimization of the microbiological quality control validation of corneal medium using a clinical C. acnes isolate
Source: Cell Tissue Bank. 2026 Feb 19;27(1):12. doi: 10.1007/s10561-026-10211-9 (PMC12920408; doi:10.1007/s10561-026-10211-9)
Supplement: Supplementary file 5 — Supplementary file5 (JPG 43 KB) [file 10561_2026_10211_MOESM5_ESM.docx]

| Strain | Isolate origin | Minimum inhibitory concentration (MIC) for penicillin | First experiment setup: time-to-detection (TTD) in NaCl 0,9% | Second experiment setup: TTD in NaCl 0,9% vs. TTD in cornea organ culture medium (cocm) | Third experiment setup: TTD in NaCl 0,9% vs. TTD in cocm |
| --- | --- | --- | --- | --- | --- |
| CA01 | clinical |  | No growth detected |  |  |
|  |  |  |  |  |  |
|  |  |  |  |  |  |
|  |  |  |  |  |  |
|  |  |  |  |  |  |
| **CA02^a^** | clinical | 0,032 μg/mL | 212.2 h | Medium: 282.23 h  NaCl: 223.22 h | Medium: 327 h  NaCl: 245 h |
|  |  |  | 206 h | Medium: 269.68 h  NaCl: 246.68 h | Medium: 250 h  NaCl: 295 h |
|  |  |  | 208.1 h | Medium: 247.6 h  NaCl: 243.08 h | Medium: 282 h  NaCl: 228 h |
|  |  |  | 229.3 h | Medium: 257.82 h  NaCl: 226.32 h | Medium: 262 h  NaCl: 225 h |
|  |  |  | 208 h | Medium: 257.12 h  NaCl: 221.03 h | Medium: 270 h  NaCl: 227 h |
| CA03 | clinical | 0,064 μg/mL | 193.5 h | Medium: 257.4 h  NaCl: 174.63 h |  |
|  |  |  | 254.7 h | Medium: 257.02 h  NaCl: 182.07 h |  |
|  |  |  | 175.5 h | Medium: No growth detected  NaCl: 175.45 h |  |
|  |  |  | 179.3 h | Medium: No growth detected  NaCl: 178.87 h |  |
|  |  |  | 189.6 h | Medium: 287.47 h  NaCl: 177.83 h |  |
| CA04 | clinical | 0,032 μg/mL | 249.5 h | Medium: 289.62 h  NaCl: 221.88 h |  |
|  |  |  | 227.5 h | Medium: 268.52 h  NaCl: 207.1 h |  |
|  |  |  | 240.1 h | Medium: No growth detected  NaCl: 223.6 h |  |
|  |  |  | 239.1 h | Medium: No growth detected  NaCl: 223.82 h |  |
|  |  |  | 256.2 h | Medium: 275.62 h  NaCl: 228.03 h |  |
| CA05 | clinical |  | No growth detected |  |  |
|  |  |  |  |  |  |
|  |  |  |  |  |  |
|  |  |  |  |  |  |
|  |  |  |  |  |  |
| CA06 | clinical |  | 285.4 h |  |  |
|  |  |  |  |  |  |
|  |  |  |  |  |  |
|  |  |  |  |  |  |
|  |  |  |  |  |  |
| CA07 | clinical |  | No growth detected |  |  |
|  |  |  |  |  |  |
|  |  |  |  |  |  |
|  |  |  |  |  |  |
|  |  |  |  |  |  |
| CA08 | clinical |  | No growth detected |  |  |
|  |  |  |  |  |  |
|  |  |  |  |  |  |
|  |  |  |  |  |  |
|  |  |  |  |  |  |
| CA09 | clinical |  | No growth detected |  |  |
|  |  |  |  |  |  |
|  |  |  |  |  |  |
|  |  |  |  |  |  |
|  |  |  |  |  |  |
| CA10 | clinical | 0,016 μg/mL | 311.3 h | Medium: 300.43 h  NaCl: 260.55 h |  |
|  |  |  | 323 h | Medium: 322.08 h  NaCl: 278.33 h |  |
|  |  |  | 335.1 h | Medium: 309.67 h  NaCl: 262.97 h |  |
|  |  |  | 297.1 h | Medium: 294.27 h  NaCl: 273.1 h |  |
|  |  |  | 273.5 h | Medium: 299.97 h  NaCl: 265.77 h |  |
| CA11 | clinical |  | 300.3 h |  |  |
|  |  |  |  |  |  |
|  |  |  |  |  |  |
|  |  |  |  |  |  |
|  |  |  |  |  |  |
| CA12 | clinical | 0,032 μg/mL | 333 h | Medium: No growth detected  NaCl: No growth detected |  |
|  |  |  | 333.3 h | Medium: No growth detected  NaCl: No growth detected |  |
|  |  |  | No growth detected | Medium: No growth detected  NaCl: No growth detected |  |
|  |  |  | 309.3 h | Medium: No growth detected  NaCl: No growth detected |  |
|  |  |  | 288.6 h | Medium: No growth detected  NaCl: 329.62 h |  |
| CA13 | clinical |  | No growth detected |  |  |
|  |  |  |  |  |  |
|  |  |  |  |  |  |
|  |  |  |  |  |  |
|  |  |  |  |  |  |
| CA14 | clinical | 0,016 μg/mL | 241.9 h | Medium: No growth detected  NaCl: 311.63 h |  |
|  |  |  | 179.3 h | Medium: No growth detected  NaCl: 301.37 h |  |
|  |  |  | 260.8 h | Medium: No growth detected  NaCl: 266.68 h |  |
|  |  |  | 260.4 h | Medium: No growth detected  NaCl: 278.17 h |  |
|  |  |  | 261.8 h | Medium: No growth detected  NaCl: 285.63 h |  |
| CA15 | clinical |  | 299.2 h |  |  |
|  |  |  |  |  |  |
|  |  |  |  |  |  |
|  |  |  |  |  |  |
|  |  |  |  |  |  |
| CA16 | clinical |  | 285.4 h |  |  |
|  |  |  |  |  |  |
|  |  |  |  |  |  |
|  |  |  |  |  |  |
|  |  |  |  |  |  |
| CA17 | clinical |  | No growth detected |  |  |
|  |  |  |  |  |  |
|  |  |  |  |  |  |
|  |  |  |  |  |  |
|  |  |  |  |  |  |
| CA18 | clinical |  | No growth detected |  |  |
|  |  |  |  |  |  |
|  |  |  |  |  |  |
|  |  |  |  |  |  |
|  |  |  |  |  |  |
| CA19 | clinical |  | No growth detected |  |  |
|  |  |  |  |  |  |
|  |  |  |  |  |  |
|  |  |  |  |  |  |
|  |  |  |  |  |  |
| CA20 | skin |  | 321.1 h |  |  |
|  |  |  | No growth detected |  |  |
|  |  |  | No growth detected |  |  |
|  |  |  | No growth detected |  |  |
|  |  |  | No growth detected |  |  |
| CA21 | skin |  | 297.9 h |  |  |
|  |  |  |  |  |  |
|  |  |  |  |  |  |
|  |  |  |  |  |  |
|  |  |  |  |  |  |
| CA22 | skin | 0,032 μg/mL | 164.3 h | Medium: 256.58 h  NaCl: 232.57 h |  |
|  |  |  | 190.3 h | Medium: 246.37 h  NaCl: 209.12 h |  |
|  |  |  | 202.4 h | Medium: 259.35 h  NaCl: 212.47 h |  |
|  |  |  | 207.9 h | Medium: 255.5 h  NaCl: 200.67 h |  |
|  |  |  | 187.9 h | Medium: 290.33 h  NaCl: 207.17 h |  |
| CA23 | skin |  | 306.1 h |  |  |
|  |  |  | 287.6 h |  |  |
|  |  |  | 324.9 h |  |  |
|  |  |  | 274.6 h |  |  |
|  |  |  | 204.2 h |  |  |
| ATCC 11827 | skin (probably) | 0,016 μg/mL |  | Medium: No growth detected NaCl: No growth detected | Medium: No growth detected  NaCl: No growth detected |
|  |  |  |  | Medium: No growth detected NaCl: 196.95 h | Medium: 211.28  NaCl: 148.08 |
|  |  |  |  | Medium: No growth detected NaCl: 209.63 hhhhh | Medium: No growth detected  NaCl: 330.5 |
|  |  |  |  | Medium: 268.35 h  NaCl: 187.22 h | Medium: No growth detected  NaCl:328 |
|  |  |  |  | Medium: No growth detected NaCl: 195.52 h | Medium: No growth detected  NaCl: 133 |

^a^This isolate was deposited as *C. acnes* DSM 117854 at DSMZ
